# Supplementary material for: Determining the Benzo[a]pyrene Degradation, Tolerance, and Adsorption Mechanisms of Kefir-Derived Bacterium Bacillus mojavensis TC-5
Source: Foods. 2025 Aug 4;14(15):2727. doi: 10.3390/foods14152727 (PMC12346863; doi:10.3390/foods14152727)
Supplement: Supplementary file 1 [file foods-14-02727-s001.zip › foods-3768383-supplementary.pdf]

Supplementary material

Determining the Benzo[a]pyrene Degradation, Tolerance, and Adsorption Mechanisms of Kefir-Derived Bacterium *Bacillus mojavensis* TC-5

Zhixian Duo [1,2,†], Haohao Li [1,2,†], Zeyu Wang [1,2], Zhiwei Zhang [3], Zhuonan Yang [4], Aofei Jin [4], Minwei Zhang [1], Rui Zhang [4,*], Yanan Qin [1,2,*]

1. Smart Agriculture College of Xinjiang University (Research Institute), Xinjiang University, Urumqi, 830017, China;

2. Xinjiang Key Laboratory of Biological Resources and Genetic Engineering, Xinjiang University, Urumqi, 830046, China;

3. Institute of Materia Medica, Xinjiang University, Urumqi, 830046, China;

4. Xinjiang Key Laboratory of Special Species Conservation and Regulatory Biology, College of Life Sciences, Xinjiang Normal University, Urumqi, 830054, China;

* Corresponding Author: qin@xju.edu.cn; zhangrui1124@xjnu.edu.cn

[†] Those authors equally contributed to this paper.

Directory

Material and methods

Medium formulation

BaP biodegradation analysis

Figure S1 BaP standard curve.

Table S1 Single-factor experiment levels.

Table S2 Orthogonal experiment factors and levels.

Table S3 Orthogonal experiment results and analysis.

Degradation products analysis—GC-MS

Results and discussion

Figure S2 Phylogenetic tree based on 16S rRNA gene sequence of *Bacillus mojavensis* TC-5. (Neighbor-joining,)

Figure S3 Degradation efficiency and OD_{600} value of TC-5 at different BaP concentrations.

Figure S4 Degradation efficiency and OD_{600} value of TC-5 at different pH values.

Figure S5 Degradation efficiency and OD_{600} value of TC-5 at different rotational speeds.

Figure S6 Chromatogram data before and after optimization. (a) Unoptimized degradation group; (b) optimized degradation group; (c) optimized removal group.

Figure S7 Genome map of the chromosome of *Bacillus mojavensis* TC-5.

Figure S8 The KEGG annotation of the *Bacillus mojavensis* TC-5 genome.

Figure S9 GC-MS total ion flow diagram of BaP degradation products by TC-5.

Figure S10 GC-MS diagram of degradation products of TC-5.

Table S4 *Bacillus mojavensis* TC-5 genome basic information.

Table S5 Information on genes related to BaP degradation, adsorption, and tolerance.

Table S6 Information on strain TC-5 versus the remaining BaP-degrading strains.

Material and methods

Medium formulation

MRS (liquid): 10 g peptone, 10 g beef extract powder, 5 g yeast extract fermentation, 2 g KH_2PO_4, 2 g $C_6H_{14}N_2O_7$, 5 g CH_3COONa, 20 g glucose, 0.58 g $MgSO_4·7H_2O$, 0.25 g $MnSO_4·4H_2O$, 1 mL Tween 80, and 1000 mL distilled water, pH = 6.2- 6.4, 115 °C, autoclave for 20 min.

MRS (solid): 10 g peptone, 10 g beef extract powder, 5 g yeast extract fermentation, 2 g KH_2PO_4, 2 g $C_6H_{14}N_2O_7$, 5 g CH_3COONa, 20 g glucose, 0.58 g $MgSO_4·7H_2O$, 0.25 g $MnSO_4·4H_2O$, 1 mL Tween 80, 18 g agar, and 1000 mL distilled water, pH = 6.2- 6.4, 115 °C, autoclave for 20 min.

BaP biodegradation analysis

The HPLC conditions were as follows: C18 Diamosil TM reversed-phase column (4.6 mm × 150 mm × 3.5 μm); mobile phase: pure methanol/water (100/0, volume ratio); UV detector: wavelength 245 nm; injection volume: 10 μL; column temperature: 39 °C; retention time: 8 min; detection flow rate: 1 mL/min. BaP standard solutions were prepared in six concentration gradients, and the peak areas were determined. The standard curve of BaP was plotted using the software origin 2021 with BaP concentration as the abscissa and peak area as the ordinate. The linear equation of the standard curve is y= 80.5036 X + 25.4214, and the correlation coefficient R^2 = 0.9996. The reagent blank group and sample blank control group were installed in this test, respectively.

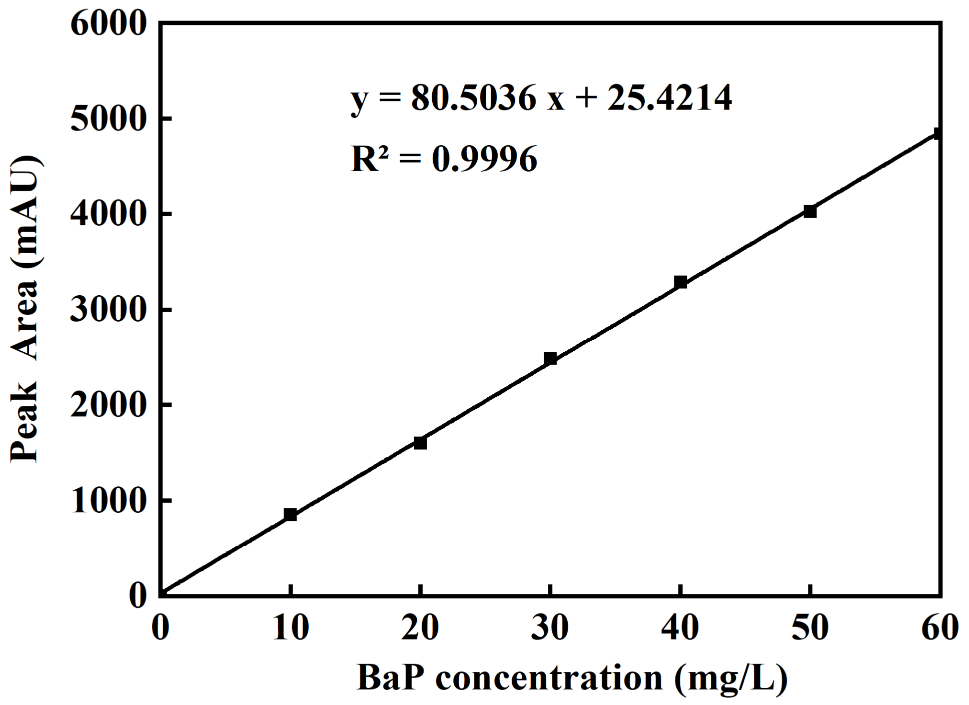

Figure S1 BaP standard curve.

Table S1 Single-factor experiment levels.

Factors	Fixed condition	Variable condition
BaP concentration（mg/L）	pH：6；rotational speed：140	BaP concentration：10、15、20、25、30
pH value	BaP concentration：20；rotational speed：140	pH：4、5、6、7、8
rotational speed（rpm）	BaP concentration：20；pH：6	rotational speed：120、140、160、180、200

Table S2 Orthogonal experiment factors and levels.

Levels	Factors		
	A: BaP concentration /mg. L^{-1}	B: pH value	C: rotational speed / rpm
1	15	6	140
2	20	6.5	160
3	25	7	180

Table S3 Orthogonal experiment results and analysis.

Trial No.	A	B	C	Degradation efficiency (%)
	BaP concentration /mg. L^{-1}	pH value	Rotational speed / rpm	
1	1	1	1	14.89
2	1	2	2	12.48
3	1	3	3	16.78
4	2	1	2	18.78
5	2	2	3	22.23
6	2	3	1	21.86
7	3	1	3	18.58
8	3	2	1	20.53
9	3	3	2	24.95
K1	44.151	52.251	57.279	
K2	62.871	55.239	56.211	
K3	64.059	63.591	57.591	
k1	14.717	17.417	19.093	
k2	20.957	18.413	18.737	
k3	21.25353	21.197	19.197	
R	6.636	3.780	0.460	

Degradation products analysis—GC-MS

The GC-MS conditions were as follows: the chromatographic column was HP-5MS (30.0 m × 250 μm, 0.25 μm); the temperature of the inlet port was 250 ℃. The warming procedure was as follows: the starting temperature was 80 ℃ and kept for 1 min; the temperature was warmed up to 180 ℃ by 10 ℃/min and kept for 5 min; the temperature was warmed up to 280 ℃ by 8 ℃/min and kept for 5 min; the temperature was warmed up to 300 ℃ by 10 ℃/min and kept for 3 min; the carrier gas was helium with a flow rate of 1.0 mL/min and the sample was injected without diversion; and the conditions of MS were as follows: EI source, electron energy of 70 eV; transmission line temperature of 250 ℃; ion source temperature of 230 ℃; quadrupole temperature of 150 ℃; mass scanning range of 35 u~650 u; scanning mode: full scanning; solvent delay of 3 min. Detection of the components was performed using the MS database NIST14 and retention time.

Results and discussion

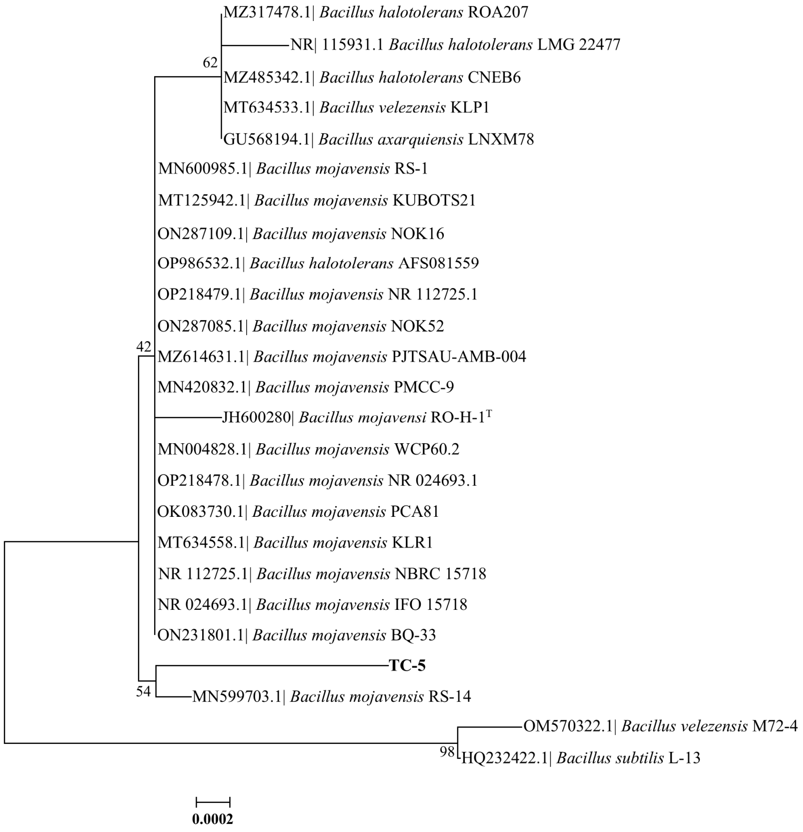

Figure S2 Phylogenetic tree based on 16S rRNA gene sequence of *Bacillus mojavensis* TC-5. (Neighbor-joining.)

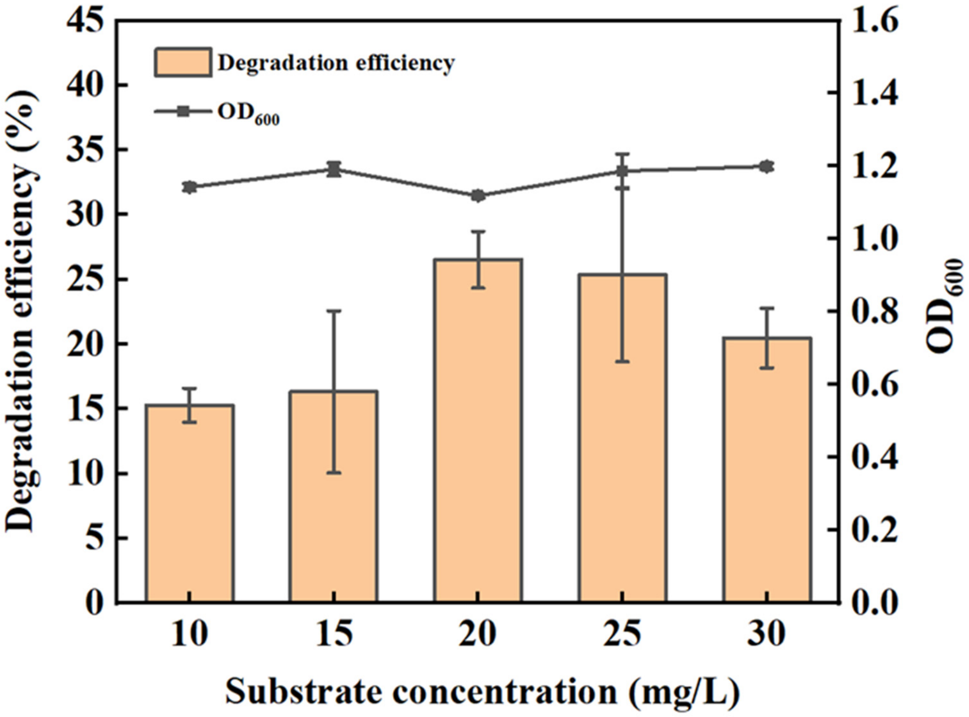

Figure S3 Degradation efficiency and OD_{600} value of TC-5 at different Bap concentrations.

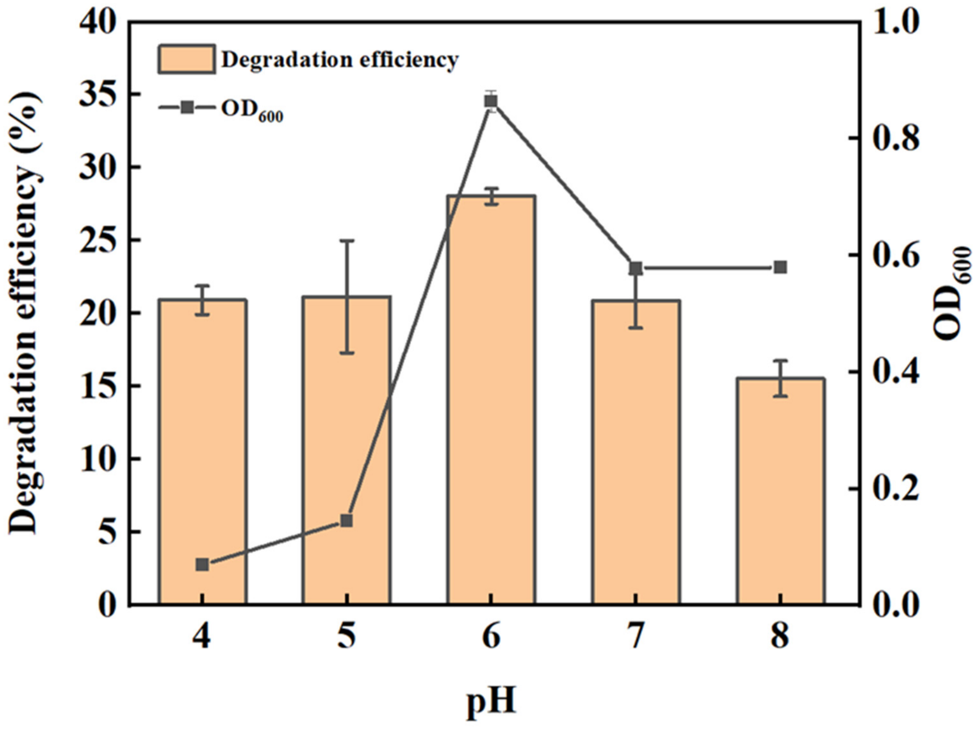

Figure S4 Degradation rate and OD_{600} value of TC-5 at different pH values.

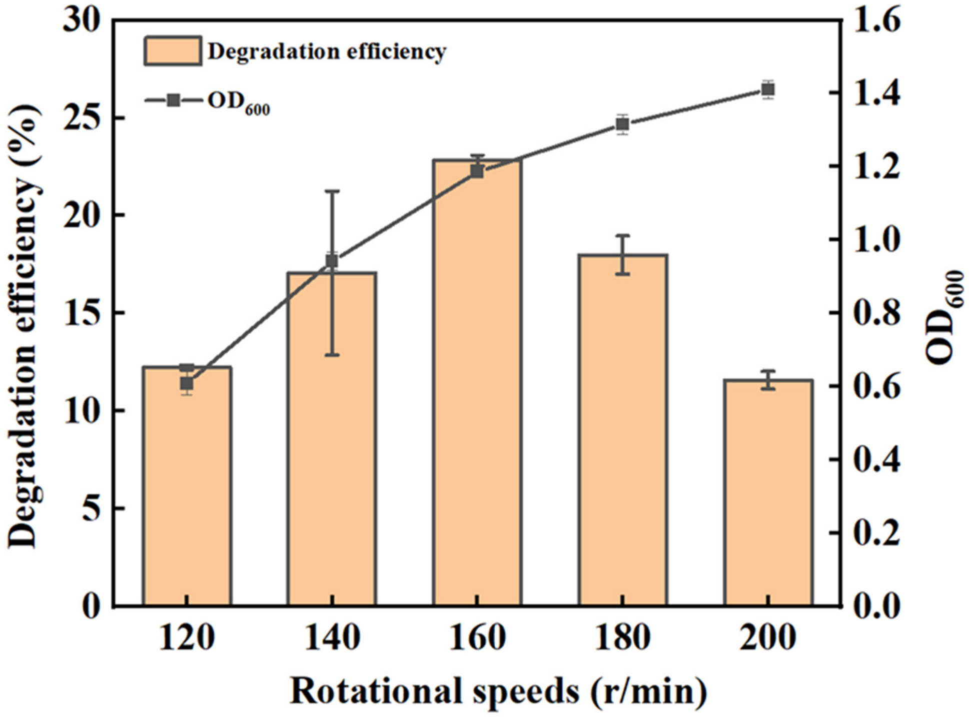

Figure S5 Degradation efficiency and OD_{600} value of TC-5 at different rotational speeds.

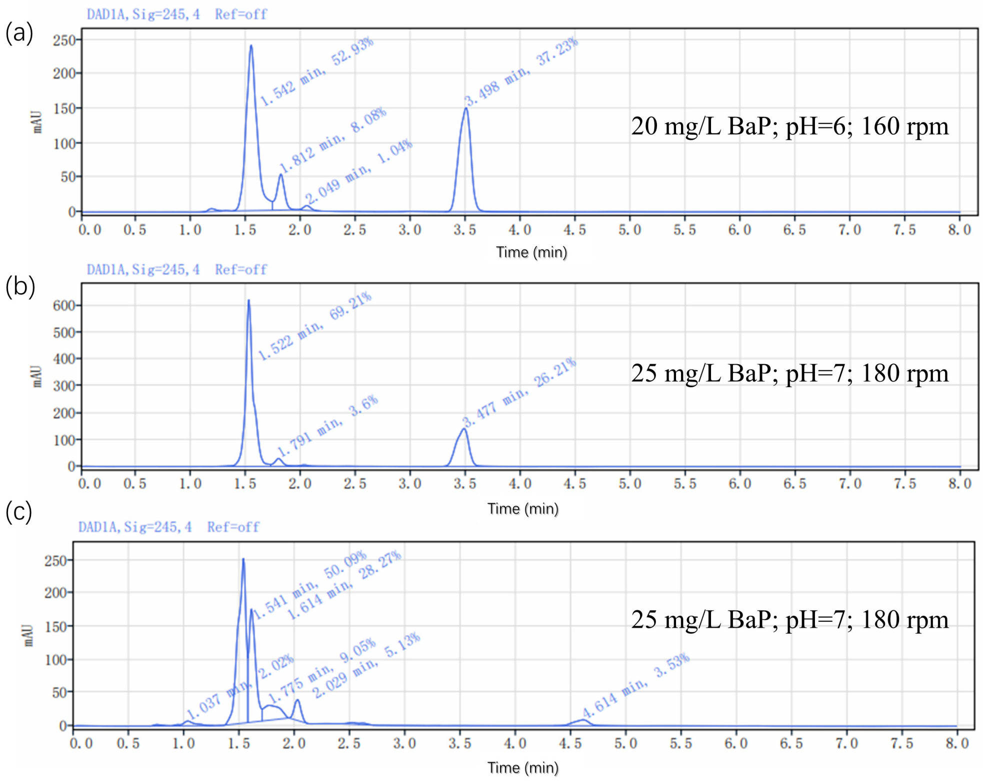

Figure S6 Chromatogram data before and after optimization. (a) Unoptimized degradation group; (b) optimized degradation group; (c) optimized removal group.

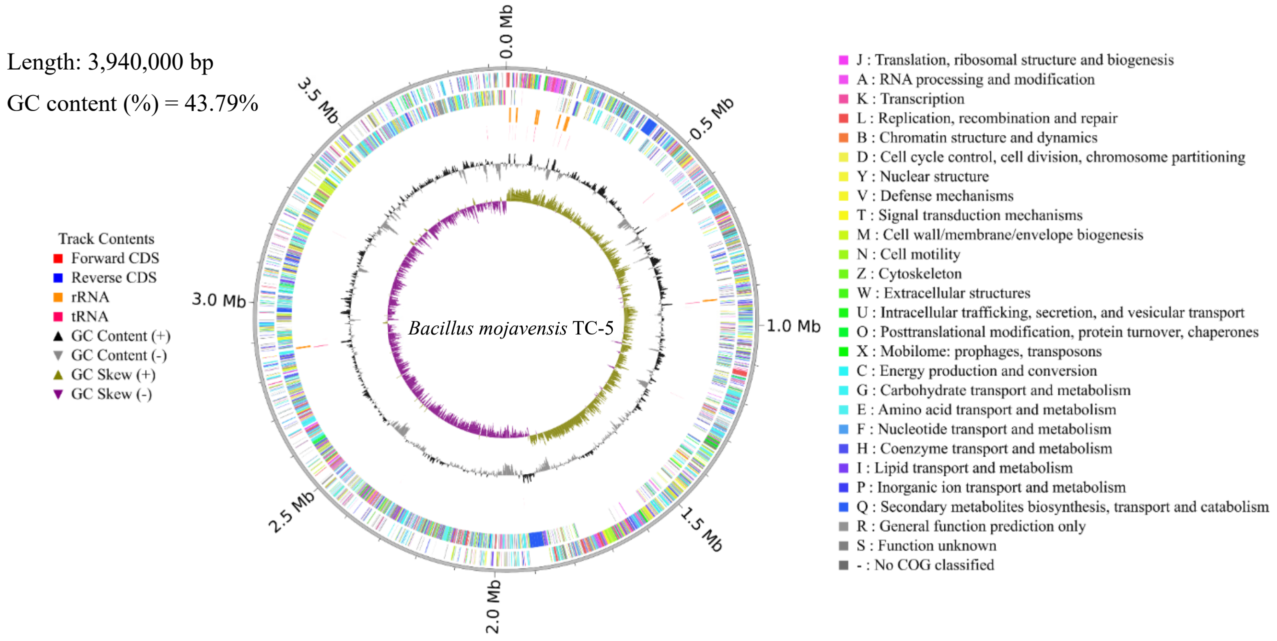

Figure S7 Genome map of the chromosome of *Bacillus mojavensis* TC-5. From the outside to inside: the first circle denotes the scale; the second and third circles are the genes in the positive and negative strands of the genome; and the different colors represent different COG functional classifications. The fourth and fifth circles are for rRNA and tRNA, pink for tRNA, and orange for rRNA; the sixth circle is the GC content (peaks out/inside the circle indicate values higher or lower than average G + C content, respectively); the innermost circle is the GC skew (calculated as (G − C)/(G + C), green/purple peaks out/inside the circle indicates values higher or lower than 1, respectively).

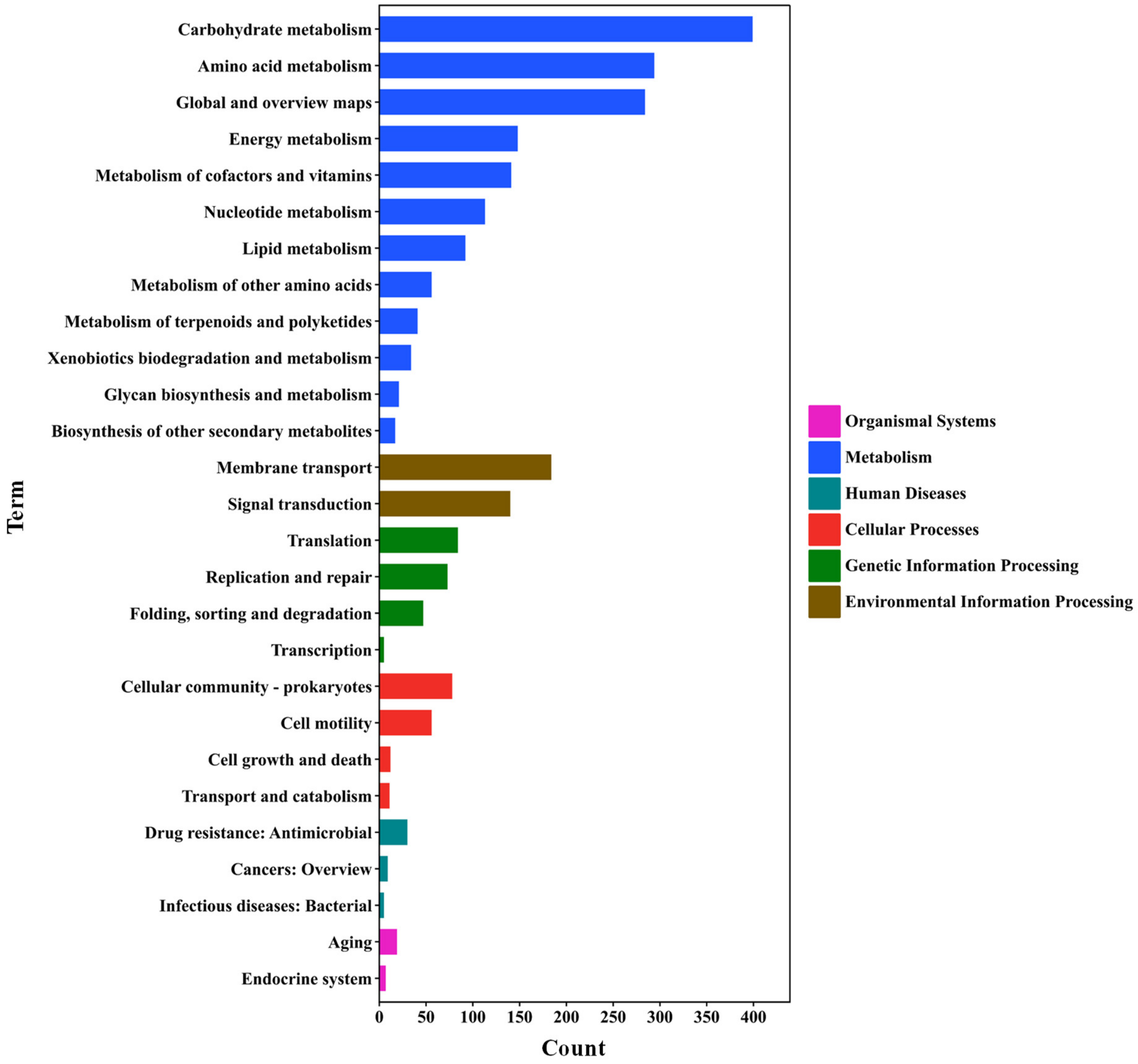

Figure S8 The KEGG annotation of the *Bacillus mojavensis* TC-5 genome.

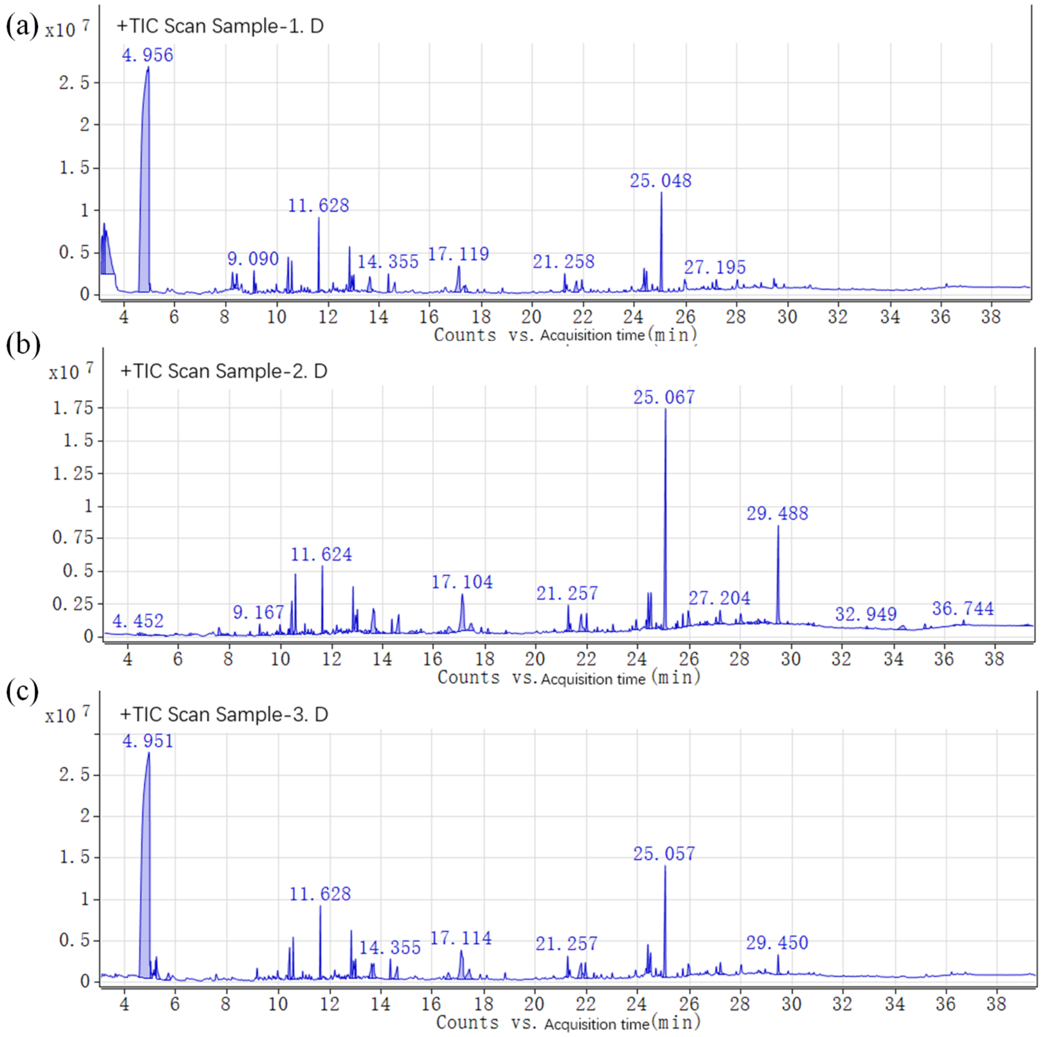

Figure S9 GC-MS total ion flow diagram of BaP degradation products byTC-5. (a) Group 1 with no BaP added and TC-5 added; (b) Group 2 with no TC-5 added and BaP added; (c) Group 3 with both TC-5 and BaP added.

(a)

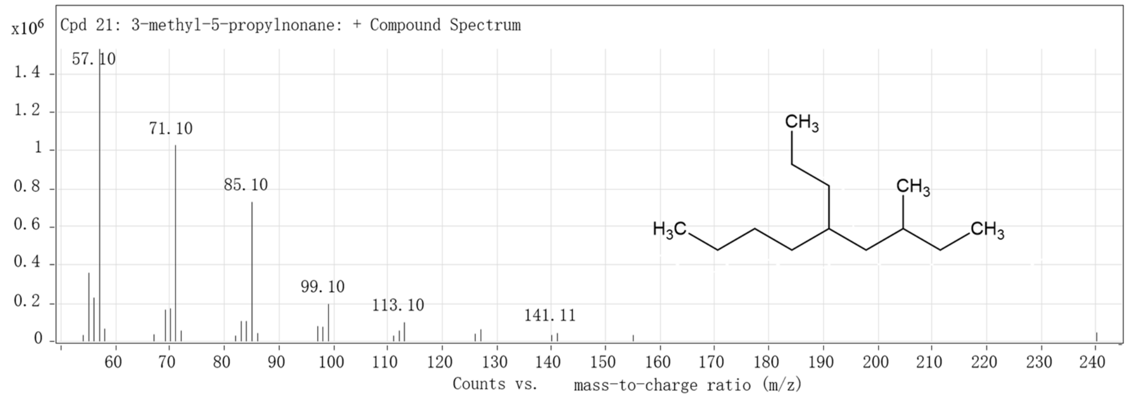

(b)

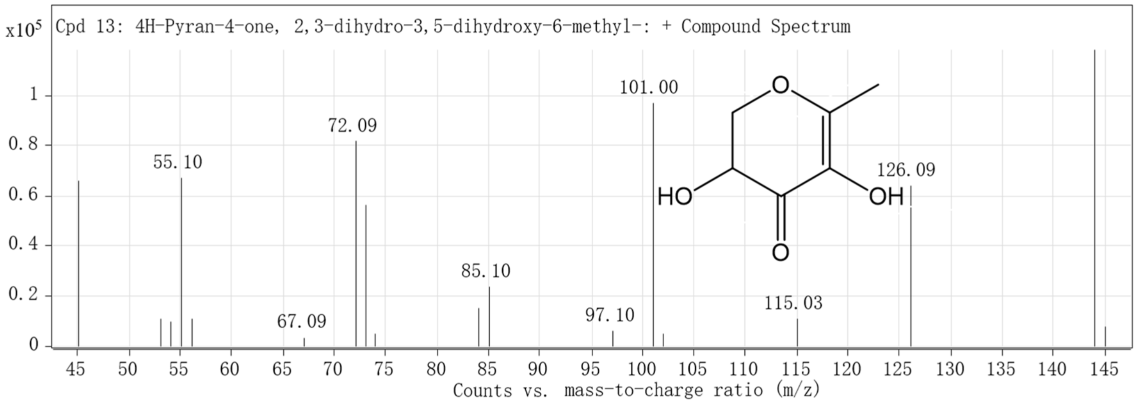

Figure S10 GC-MS diagram of degradation products of TC-5. (a) 3-methyl-5-propylnonane; (b) 4H-Pyran-4-one, 2,3-dihydro-3,5-dihydroxy-6-methyl.

Table S4 *Bacillus mojavensis* TC-5 genome basic information.

Attribute	*B. mojavensis* TC-5
Length (bp)	3,940,000
GC content (%)	43.79
Gene/genome (%)	88
Total bases (bp)	1,045,364,669
Total reads	12,340,160
Reads number	389,499
Reads N50 (bp)	3,914
Average length (bp)	867
Protein coding genes	3911
No. of ribosomal RNA	5
No. of transfer RNA	86

Table S5 Information on genes related to BaP degradation, adsorption, and tolerance.

Functional classification	Protein id	Position	Gene name	Size/aa	Strand	KO Name	Predicted function	Closest relatives			
								Organism	Class	Identity	Accession no.
Degradation	AAEQ98_01545	276404..277870	*gucD*	488	+	ko00620	alpha-ketoglutaric semialdehyde dehydrogenase GucD	*Burkholderia vietnamiensis* strain G4	Betaproteobacteria	32.98%	ABO56324.1
	AAEQ98_01705	305919..306851	*iolS_1*	310	+	0	aldo/keto reductase	*Burkholderia vietnamiensis* strain G4	Betaproteobacteria	32.91%	ABO56739.1
	AAEQ98_01820	331133..332389	*opuAA*	418	+	ko02010	glycine/proline betaine ABC transporter ATP-binding protein OpuAA	*Burkholderia vietnamiensis* strain G4	Betaproteobacteria	50.16%	ABO56460.1
	AAEQ98_01840	335436..336869		477	+	0	YfcC family protein	*Bacillus subtilis* SR1	Bacilli	25.45%	ASC82817.1
	AAEQ98_01935	353768..355315	*pruA*	515	+	ko00250	L-glutamate gamma-semialdehyde dehydrogenase	*Burkholderia vietnamiensis* strain G4	Betaproteobacteria	35.14%	ABO53145.1
	AAEQ98_02170	421605..422348	*tcyC*	247	-	ko02010	cystine ABC transporter ATP-binding protein TcyC	*Burkholderia vietnamiensis* strain G4	Betaproteobacteria	49.39%	ABO58510.1
	AAEQ98_02175	422362..423066	*tcyB*	234	-	ko02010	cystine ABC transporter permease TcyB	*Burkholderia vietnamiensis* strain G4	Betaproteobacteria	32.69%	ABO58509.1

AAEQ98_02345	455327..456715	*gabD*	462	+	ko00250	succinate-semialdehyde dehydrogenase	*Burkholderia vietnamiensis* strain G4	Betaproteobacteria	30.87%	ABO56324.1
AAEQ98_02475	478798..480882	*mtlR*	694	+	0	PRD domain-containing protein	*Burkholderia vietnamiensis* strain G4	Betaproteobacteria	36.59%	ABO53529.1
AAEQ98_03930	770434..771543	*hpxO*	369	+	0	FAD-dependent oxidoreductase	*Acinetobacter* XS-4	Gammaproteobacteria	25.69%	WP_252612560.1
AAEQ98_03955	774724..777912	*cypD*	1062	+	ko00071	bifunctional cytochrome P450/NADPH--P450 reductase	*Pontibacillus* sp. HN14	Bacilli	59.12%	WP_231415692.1
AAEQ98_04005	789162..790619	*yfmT*	485	+	ko00071	benzaldehyde dehydrogenase	*Burkholderia vietnamiensis* strain G4	Betaproteobacteria	30.27%	ABO56324.1
AAEQ98_04435	881170..882027		285	+	ko00362	VOC family protein	*Bacillus subtilis* SR1	Bacilli	89.47%	ASC83830.1
AAEQ98_04585	907585..908448	*ephA*	287	+	0	alpha/beta hydrolase	*Altererythrobacter epoxidivorans* CGMCC 1.7731^T	Alphaproteobacteria	25.93%	ALE17564.1
AAEQ98_05175	1011381..1012388	*yhdN*	335	+	0	aldo/keto reductase	*Burkholderia vietnamiensis* strain G4	Betaproteobacteria	30.74%	ABO56739.1
AAEQ98_05520	1076556..1077533	*fabHB*	326	-	ko01212	beta-ketoacyl-ACP synthase III FabHB	*Bacillus licheniformis* M2-7	Bacilli	71.91%	WP_011197721.1
AAEQ98_05570	1084774..1086315	*lcfB*	513	+	ko02024	fatty acid--CoA ligase family protein	*Burkholderia vietnamiensis* strain G4	Betaproteobacteria	30.06%	ABO53528.1

AAEQ98_05685	1106736..1108235	*otcC*	499	+	0	monooxygenase	*Bacillus licheniformis* M2-7	Bacilli	27.11%	WP_011198399.1
AAEQ98_06115	1191803..1192741	*fabH*	312	+	ko01212	beta-ketoacyl-ACP synthase III	*Bacillus licheniformis* M2-7	Bacilli	40.06%	WP_011197721.1
AAEQ98_06635	1280255..1281445	*yjiB_1*	396	+	0	cytochrome P450	*Bacillus thuringiensis* GIMCC1.817	Bacilli	48.63%	ART94430.1
AAEQ98_07090	1361265..1362467	*hmpA*	418	+	0	NO-inducible flavohemoprotein	*Burkholderia vietnamiensis* strain G4	Betaproteobacteria	26.32%	ABO53321.1
AAEQ98_08370	1605167..1606078	*rluD_2*	303	+	0	RluA family pseudouridine synthase	*Leptospira borgpetersenii serovar Hardjo-bovis* str. L550		23.75%	WP_002754960.1
AAEQ98_08595	1652476..1653216	*fabG*	246	+	ko01212	3-oxoacyl-[acyl-carrier-protein] reductase	*Altererythrobacter epoxidivorans* CGMCC 1.7731^T	Alphaproteobacteria	28.8%	ALE15849.1
AAEQ98_09030	1736381..1737253	*dapA*	290	+	ko01230	4-hydroxy-tetrahydrodipicolinate synthase	*Bacillus cereus* AH1271	Bacilli	59.31%	ZP_04187429.1
							Burkholderia sp. Ch1-1	Betaproteobacteria	29.66%	EIF28466
							Rhodococcus opacus R7	Actinobacteria	26.71%	AII11501
AAEQ98_09750	1863836..1864978	*bcd*	380	-	0	acyl-CoA dehydrogenase	*Burkholderia vietnamiensis* strain G4	Betaproteobacteria	46.83%	ABO56326.1
AAEQ98_09850	1916401..1917882	*gltD*	493	-	ko00910	glutamate synthase small subunit	*Burkholderia vietnamiensis* strain G4	Betaproteobacteria	48.43%	ABO53398.1
AAEQ98_09855	1917899..1922461	*gltB*	1520	-	ko00910	glutamate synthase large subunit	*Burkholderia vietnamiensis* strain G4	Betaproteobacteria	50.45%	ABO53397.1

AAEQ98_04155	821264..821539	*acyP*	91	-	ko00627	acylphosphatase	-	-	-	-
AAEQ98_09880	1926393..1927610	*yjiB_2*	405	+	0	cytochrome P450	*Bacillus thuringiensis* GIMCC1.817	Bacilli	35.63%	ART94430.1
							Altererythrobacter epoxidivorans CGMCC 1.7731T	Alphaproteobacteria	30.92%	ALE17673.1
AAEQ98_10215	1985592..1986344		250	-	0	GNAT family N-acetyltransferase	*Acinetobacter* XS-4	Gammaproteobacteria	31.48%	WP_252612828.1
AAEQ98_03160	605127..606515	*phoB*	462	-	ko00627	alkaline phosphatase	-	-	-	-
AAEQ98_10335	2005305..2006792	*dhaS*	495	+	ko00071	aldehyde dehydrogenase DhaS	*Burkholderia vietnamiensis* strain G4	Betaproteobacteria	35.62%	ABO56324.1
							Burkholderia vietnamiensis strain G4	Betaproteobacteria	29.98%	ABO59673.1
AAEQ98_11705	2239239..2240018		259	-	0	SDR family oxidoreductase	*Altererythrobacter epoxidivorans* CGMCC 1.7731^{T}	Alphaproteobacteria	31.49%	ALE15849.1
AAEQ98_01630	291360..293111	*phoD*	583	+	ko00627	alkaline phosphatase D	-	-	-	-
AAEQ98_11745	2247381..2248850	*zwf*	489	+	ko01200	glucose-6-phosphate dehydrogenase	*Acinetobacter* XS-4	Gammaproteobacteria	43.24%	WP_252612827.1

AAEQ98_11810	2259410..2260132	*artR*	240	-	0	arginine ABC transporter ATP-binding protein ArtR	*Burkholderia vietnamiensis* strain G4	Betaproteobacteria	51.22%	ABO58510.1
AAEQ98_11815	2260125..2260784	*artQ*	219	-	0	arginine ABC transporter permease ArtQ	*Burkholderia vietnamiensis* strain G4	Betaproteobacteria	28.11%	ABO58508.1
AAEQ98_11820	2260864..2261631	*artP*	255	-	0	arginine ABC transporter substrate-binding protein ArtP	*Burkholderia vietnamiensis* strain G4	Betaproteobacteria	32.74%	ABO56609.1
AAEQ98_11905	2279646..2280785	*mmgC*	379	-	0	acyl-CoA dehydrogenase	*Burkholderia vietnamiensis* strain G4	Betaproteobacteria	50.81%	ABO56326.1
AAEQ98_12820	2436481..2437440		319	-	ko01212	nitronate monooxygenase	*Altererythrobacter epoxidivorans* CGMCC 1.7731^T	Alphaproteobacteria	23.15%	ALE16332.1
AAEQ98_13135	2490941..2494105	*cypB*	1054	-	ko00627	bifunctional cytochrome P450/NADPH--P450 reductase CypB	*Pontibacillus* sp. HN14	Bacilli	58.55%	WP_231415692.1
							Altererythrobacter epoxidivorans CGMCC 1.7731^T	Alphaproteobacteria	26.44%	ALE17510.1
AAEQ98_14610	2794108..2795292	*bioI*	394	-	ko00780	cytochrome P450	*Bacillus thuringiensis* GIMCC1.817	Bacilli	30.73%	ART94430.1
AAEQ98_15130	2881558..2883030	*betB*	490	-	ko00260	betaine-aldehyde dehydrogenase	*Burkholderia vietnamiensis* strain G4	Betaproteobacteria	31.34%	ABO56324.1
AAEQ98_15340	2926403..2927227	*carC*	274	+	0	alpha/beta hydrolase	*Altererythrobacter epoxidivorans* CGMCC 1.7731^T	Alphaproteobacteria	24.58%	ALE15876.1

AAEQ98_15870	3033659.. 3034219	*pucE*	186	-	0	xanthine dehydrogenase subunit E	*Altererythrobacter epoxidivorans* CGMCC 1.7731^T	Alphaproteobacteria	42.86%	ALE17350.1
AAEQ98_15875	3034210.. 3036447	*pucD*	745	-	0	xanthine dehydrogenase subunit D	*Altererythrobacter epoxidivorans* CGMCC 1.7731^T	Alphaproteobacteria	27.35%	ALE17513.1
AAEQ98_16010	3060191.. 3061216	*metN*	341	-	ko02010	methionine ABC transporter ATP-binding protein MetN	*Burkholderia vietnamiensis* strain G4	Betaproteobacteria	38.00%	ABO58510.1
AAEQ98_16045	3063969.. 3065753	*fadE*	594	-	0	acyl-CoA dehydrogenase FadE	*Burkholderia vietnamiensis* strain G4	Betaproteobacteria	34.67%	ABO56326.1
AAEQ98_16585	3172110.. 3173255	*opuBA*	381	-	ko02010	choline ABC transporter ATP-binding protein OpuBA	*Burkholderia vietnamiensis* strain G4	Betaproteobacteria	32.50%	ABO56460.1
AAEQ98_16610	3176398.. 3177537	*opuCA*	379	-	ko02010	osmoprotectant ABC transporter ATP-binding protein OpuCA	*Burkholderia vietnamiensis* strain G4	Betaproteobacteria	32.21%	ABO56460.1
AAEQ98_19870	3829897.. 3830910	*qdoI*	337	-	0	quercetin 2,3-dioxygenase	-	-	-	-
AAEQ98_04825	941854..942990	*ssuD*	378	+	ko00920	alkanesulfonate monooxygenase	-	-	-	-
AAEQ98_15170	2890415.. 2890900	*cdoA*	161	+	ko00270	cysteine dioxygenase family protein	-	-	-	-

AAEQ98_17230	3298098..3299318	*cypX*	406	-	0	cytochrome P450, cyclodipeptide synthase-associated	*Bacillus* thuringiensis GIMCC1.817	Bacilli	29.27%	ART94430.1
AAEQ98_18190	3499443..3500699	*urtA*	418	-	ko02010	urea ABC transporter substrate-binding protein	*Burkholderia vietnamiensis* strain G4	Betaproteobacteria	22.05%	ABO53118.1
							Pseudomonas entomophila L48	Gammaproteobacteria	22.39%	WP_011532595.1
AAEQ98_18450	3544006..3545145	*acdA*	379	-	0	acyl-CoA dehydrogenase AcdA	*Burkholderia vietnamiensis* strain G4	Betaproteobacteria	50.13%	ABO56326.1
AAEQ98_19655	3789521..3790270	*yxeO*	249	-	ko02010	amino acid ABC transporter ATP-binding protein	*Burkholderia vietnamiensis* strain G4	Betaproteobacteria	45.56%	ABO58510.1
AAEQ98_19675	3792309..3793634	*ntaA*	441	-	0	LLM class flavin-dependent oxidoreductase	*Rhodococcus jostii* RHA1	Actinobacteria	35.32%	WP_011595563.1
AAEQ98_19795	3814437..3815900	*iolA*	487	-	ko01200	methylmalonate–semialdehyde dehydrogenase	*Burkholderia vietnamiensis* strain G4	Betaproteobacteria	46.47%	ABO56324.1
AAEQ98_19805	3817119..3818051	*iolS_4*	310	+	0	aldo/keto reductase	*Burkholderia vietnamiensis* strain G4	Betaproteobacteria	35.45%	ABO56739.1
AAEQ98_19840	3823453..3824274	*bacC_2*	273	+	0	SDR family oxidoreductase	*Altererythrobacter epoxidivorans* CGMCC 1.7731^T	Alphaproteobacteria	24.86%	ALE15758.1
AAEQ98_20350	3922091..3923443	*ydjE*	450	+	0	MFS transporter	*Burkholderia vietnamiensis* strain G4	Betaproteobacteria	26.13%	ABO55757.1

Adsorption	AAEQ98_14035	2671741..2672466	*lytR_1*	241	-	ko02020	LytTR family DNA-binding domain-containing protein"	-	-	-	-
	AAEQ98_20370	3925468..3926226	*xth*	252	-	ko03410	exodeoxyribonuclease III	-	-	-	-
	AAEQ98_03790	738925..740658		577	+	ko02020	sensor histidine kinase	-	-	-	-
	AAEQ98_05320	1038475..1038828		117	+	0	YlbF family regulator	-	-	-	-
Tolerance	AAEQ98_06130	1195058..1196044	*appD*	328	+	ko02024	oligopeptide ABC transporter ATP-binding protein AppD	*Burkholderia vietnamiensis* strain G4	Betaproteobacteria	47.02%	ABO56688.1
	AAEQ98_06135	1196041..1197030	*appF*	329	+	ko02024	oligopeptide ABC transporter ATP-binding protein AppF	*Burkholderia vietnamiensis* strain G4	Betaproteobacteria	38.19%	ABO56688.1
	AAEQ98_06190	1209264..1210340	*oppD*	355	+	ko02010	oligopeptide ABC transporter ATP-binding protein OppD	*Burkholderia vietnamiensis* strain G4	Betaproteobacteria	48.7%	ABO56688.1
	AAEQ98_06195	1210342..1211259	*oppF*	305	+	ko02010	oligopeptide ABC transporter ATP-binding protein OppF	*Burkholderia vietnamiensis* strain G4	Betaproteobacteria	41.09%	ABO56688.1
	AAEQ98_07045	1351582..1352589	*oppD_3*	335	+	ko02010	ABC transporter ATP-binding protein	*Burkholderia vietnamiensis* strain G4	Betaproteobacteria	48.37%	ABO56688.1
	AAEQ98_07070	1357285..1358268	*oppF_3*	327	+	ko02024	oligopeptide/dipeptide ABC transporter ATP-binding protein	*Burkholderia vietnamiensis* strain G4	Betaproteobacteria	39.38%	ABO56688.1

AAEQ98_12960	2457424..2458251	*nikD*	275	+	ko02010	nickel import ATP-binding protein NikD	*Burkholderia vietnamiensis* strain G4	Betaproteobacteria	42.48%	ABO56688.1
AAEQ98_01735	313098..313874	*ycdF*	258	+	ko01200	SDR family oxidoreductase	-	-	-	-
AAEQ98_10840	2092039..2092629	*eda*	196	-	ko00630	bifunctional 4-hydroxy-2-oxoglutarate aldolase/2-dehydro-3-deoxy-phosphogluconate aldolase	-	-	-	-
AAEQ98_10845	2092632..2093606	*kdgK*	324	-	ko01200	2-dehydro-3-deoxygluconokinase	-	-	-	-
AAEQ98_11755	2249629..2251038	*gndA*	469	-	ko01200	NADP-dependent phosphogluconate dehydrogenase	-	-	-	-
AAEQ98_18410	3535838..3536803	*glpX*	321	-	ko01200	class II fructose-bisphosphatase	-	-	-	-
AAEQ98_19730	3800058..3801926	*yxdM*	622	-	ko02020	ABC transporter permease YxdM	-	-	-	-
AAEQ98_04460	886485..887642		385	+	0	ABC transporter permease	-	-	-	-
AAEQ98_04455	885283..886470		395	+	0	ABC transporter permease	-	-	-	-
AAEQ98_04450	884336..885271	*yadG*	311	+	0	ABC transporter ATP-binding protein	-	-	-	-

2

Table S6 Information on strain TC-5 versus the remaining BaP-degrading strains.

Microorganisms	Sources	Key genes	Degradation time	BaP concentration	Degradation efficiency	Removal efficiency	Degradation products	References
Bacillus mojavensis TC-5	kefir grains	*fabHB, bioI, betB, qdoI, cdoA*	60 h	25 mg/L	32.89%	63.94%	4H-pyran-4-one,2,3-dihydro-3,5-dihydroxy-6-methyl, 3-methyl-5-propylnonane	-
Bacillus velezensis PMC10	fermented food	-	20 d	10 mg/L	51.32%	-	-	[18]
Lactobacillus plantarum CICC 22135	sourdough	-	4 h	10 mg/L	-	66.76%	-	[17]
Lactobacillus pentosus CICC 23163	mare's milk	-	4 h	10 mg/L	-	64.31%	-	[17]
Beijerinckia B-836	polluted stream	-	48 h	10 mg/L	-	-	cis-9,10-Dihydroxy-9,10-dihydrobenzo[a]pyrene	[7]
Pontibacillus chungwhensis HN14	mangrove sediments	*cyp102 (HN14), EH(HN14)*	6 d	10 mg/L	30%	-	4,5-epoxy-BaP, BaP-trans-4,5-dihydrodiol, 1,2-dihydroxy-phenanthrene	[9,10]
Pseudomonas benzopyrenica BaP3^T	coking plant soil	*Rhd1*	7 d	15 mg/L	40-60%	-	dihydrobenzo(a)pyrene, phenylacetic acid	[11]
Bacillus licheniformis M2-7	hot springs	*catE, fabHB*	3 h	0.2 mg/L	-	-	phthalic acid	[12]
Pseudomonas sp. WD23	petroleum industry wastewater	*C23O*	7 d	10 mg/L	90%	-	dihydroxypyrene, naphthalene-1,2-dicarboxylic acid, salicylic acid, oxalic acid	[11]
